# Prevalence and management of neuropathic injury caused by dental implant insertion in mandible: a systematic review

**DOI:** 10.22514/jofph.2024.012

**Published:** 2024-06-12

**Authors:** Jéssica Conti Réus, Patrícia Pauletto, Felipe Cechinel Veronez, Beatriz Dulcinéia Mendes Souza, Guenther Schuldt Filho, Cristine Miron Stefani, Carlos Flores-Mir, Graziela De Luca Canto

**Affiliations:** Department of Dentistry, Brazilian Centre for Evidence Based Research, Federal University of Santa Catarina, 88040-900 Florianópolis, SC, Brazil; Department of Dentistry, Federal University of Santa Catarina, 88040-900 Florianópolis, SC, Brazil; Faculty of Dentistry, University of Las Americas (UDLA), 170517 Quito, Ecuador; Department of Dentistry, University of the Extreme South of Santa Catarina, 88806-000 Criciúma, SC, Brazil; Department of Dentistry, Nova Southeastern University, Tampa Bay Campus, Clearwater, FL 33759, USA; Department of Dentistry, University of Brasília, 70910-900 Brasília, DF, Brazil; Department of Dentistry, Faculty of Medicine and Dentistry, University of Alberta, Edmonton, AB T6G 1C9, Canada

**Keywords:** Dental implant, Inferior alveolar nerve, Trigeminal nerve injuries, Neuralgia, Evidence-based dentistry

## Abstract

To synthesize scientific knowledge regarding the prevalence of neuropathies and nerve injuries caused by dental implant placement in mandible and the available management. Observational and interventional studies evaluating neuropathies occurrence in adults who underwent dental implant surgery were included. Any neuropathy diagnostic was accepted. The searches were conducted in six databases and grey literature. Methodological quality was screened using the Joanna Briggs Institute. The resulting synthesis was a narrative summary, and prevalence meta-analyses were performed in MetaXL 5.3. Among 98 full texts assessed, 38 studies were included. Neuropathies were diagnosed by questionnaires and/or clinical assessment. Eighteen studies presented high, sixteen moderate, and four low methodological quality. In implant surgeries without nerve lateralization, 12% and 5% of the patients may experience neuropathy during the first week and after three months, respectively. In implant surgeries with nerve lateralization, the prevalence was from 90% in the first week to 42% after three months. Proposed management included drugs, laser therapy and dental implant removal. In mandible, the prevalence of neuropathies in dental implant surgeries without lateralization is lower when compared with those with lateralization (eight times more in both follow-up times). The most frequent treatment was pharmacologic management.

## 1. Introduction

According to the American Academy of Orofacial Pain, orofacial pain can be defined as all pain associated with soft and/or mineralized tissues of the oral cavity and face [1]. The prevalence is higher in women (9.2%) than in men (3.8%) [2]. Orofacial pain can result from trigeminal nerve neuropathies, clinically detectable sensory deficits within the trigeminal nerve distribution (in one or more branches). Generally, it happens when the trigeminal nerves are affected directly or indirectly by another injury or dental invasive procedure, such as the dental implant placement [3, 4].

Dental implants have become a standard treatment for replacing missing teeth with a high survival rate (over 95%) [5, 6]. However, despite the idea that implant treatment can seldom fail, any intercurrence, especially pre- and intra-operative, can jeopardize the prognosis [7]. Besides patients’ health problems detected during clinical examination [8], three-dimensional imaging [9, 10, 11] must be performed before dental implant surgery. On imaging assessment, bone thickness, quality, height [12, 13] and anatomic structures, such as blood vessels and trigeminal nerve branches proximity [14, 15, 16], should be evaluated.

In the last ten years, some studies have reported the association of trigeminal nerve injury with dental implant surgery [17, 18, 19, 20, 21]. Besides published primary studies, three systematic reviews were identified. Two of them [22, 23] showed that early and correct diagnosis contributes to nerve recovery, compared to a late diagnosis, being the proportion of 93% versus 78%, respectively [22]. The third [24] reported the incidence of altered sensation after implant placement, which was higher ten days after the surgery (13%) than after one year (3%).

Knowledge of the prevalence of lesions is paramount for identifying and diagnosing these neuropathies as well as choosing the most suitable treatment. Therefore, this systematic review aims to answer two questions: (1) What is the prevalence of neuropathies or nerve injuries related to dental implant placement in mandible? and (2) What are the available current treatments for neuropathies or nerve injuries caused by dental implants?

## 2. Methods

The PEO acronym (Population, Exposure and Outcomes) was used to formulate the prevalence question, in which: (P) Adults; (E) Neuropathies or nerve injuries after dental implant placement; (O) Prevalence.

For the management question, the PICO acronym (Population, Intervention, Comparison and Outcome) was used, in which: (P) Adults with neuropathies or with nerve injured after dental implant placement; (I) Treatments; (C) Placebo or no treatment; (O) Neuropathies or nerve injuries remission.

### 2.1 Eligibility criteria

#### 2.1.1 Inclusion criteria

Observational (cross-sectional, case series and case-control) about the prevalence and interventional (randomized and non-randomized clinical trials (RCT and non-RCT), and before-and-after) studies about the management of neuropathies caused by dental implant placement were included. The study sample must have included adults (≥18 years) treated with any dental implant brand in the mandible. Any diagnostic criteria for neuropathic pain, nerve injury, treatment and follow-up time were considered. No publication year or language restrictions were applied.

#### 2.1.2 Exclusion criteria

Book chapters, conference abstracts, expert opinions, letters, literature reviews, study protocols, magazine sections, and case series with less than five patients; Non-neuropathic pain postoperative assessment; Evaluation of other outcomes than prevalence and treatment; Evaluation of neuropathic pain and/or nerve injury after procedures other than dental implant; Full-text not available or Incomplete data, even after trying to contact the corresponding authors; Presence of nerve injury and or neuropathies before the implant surgery.

### 2.2 Information sources and search strategy

The search strategy was developed with the help of an experienced health science librarian. Six electronic databases (Cochrane, Embase, LILACS, PubMed, Scopus and Web of Science) and the grey literature (on Google Scholar, OpenGrey and ProQuest Dissertation and Theses) were searched. Additionally, experts were contacted by email once per week for one month for additional studies for inclusion. Hand searches of references of included studies were also conducted. All searches were carried out on 01 December 2023. The electronic search strategy applied in the databases can be found in **Supplementary Table 1**. References were imported into a reference software manager (EndNote X9®; Bld 12062, Thomson Reuters, Philadelphia, PA, USA), and the duplicate documents were excluded.

### 2.3 Selection process

Two independent reviewers (JCR and PP) performed the selection process in two phases based on the eligibility criteria. In phase-1, titles and abstracts were screened using the online software Rayyan® (Qatar Computing Research Institute, Qatar). The studies included in phase-1 were considered in phase-2 when the full-texts were evaluated. If any disagreement arose between the first and second reviewers in any phase, a third author (FCV) was consulted to reach a final decision.

### 2.4 Data collection process

The collected data were inserted in a form previously prepared using Microsoft® Excel 16.29.1 (Microsoft Office 2019, Microsoft, Redmond, WA, USA) by the first reviewer (JCR). The second reviewer (PP) checked the data. Disagreements were resolved at a consensus meeting.

### 2.5 Data items

Data collected were the main characteristics of the study, sample, implant, neuropathy. Additional data could be added according to the goal of the included study.

### 2.6 Study methodological quality assessment

A methodological quality evaluation was performed for each included study according to its design, applying the Joanna Briggs critical (JBI) appraisal tools. Answers to each checklist item were “yes”, “no”, “unclear” or “not applicable”. A study was categorized as high methodological quality only if it had a maximum of two negative answers (“no” or “unclear”), independently of the study design [25].

### 2.7 Effect measures and synthesis methods

Primary data on the neuropathies prevalence of each included study were collected, and the meta-analysis of weighted average of the prevalence was calculated using MetaXL 5.3 software (EpiGear International Pty Ltd, Brisbane, Queensland, Australia) with a confidence interval (CI) level of 95%. Although observational (retrospective) and clinical (prospective) studies were included, they were combined in the same meta-analysis because all evaluated the patients before and after dental placement surgery.

The prevalence of neuropathies was calculated based on two-time frames: until one week of post-operative time and after three months. According to the International Classification of Orofacial Pain, the symptoms of neuropathies must be persistent or recurring for more than three months to be considered irreversible neuropathies caused by nerve lesions [3], differing from transient neuropathies (symptoms only in the first week of follow-up). A narrative summary was drafted for other outcomes to synthesize the findings and describe the identified evidence.

## 3. Results

### 3.1 Study selection

A total of 2113 studies were identified in six databases. After removing the duplicates, 1478 studies were screened in phase-1. Applying the eligibility criteria, 98 studies were eligible for the full-text evaluation in phase-2. After full-text reading, excluding 63 studies (see reasons for exclusion in **Supplementary Table 2**), and adding three studies from grey literature, 38 studies for qualitative and 30 for quantitative analyses. One included thesis [26] was also published as an article [27]; therefore, only the article was included in the quantitative analyses. A flowchart summarizing this systematic selection process is shown in Fig. 1.

**Fig. 1.** Flow diagram.

### 3.2 Study characteristics, results of individual studies and results of syntheses

#### 3.2.1 Prevalence of neuropathies after implant placement without nerve lateralization (Table 1 and **Supplementary Table 3**) [15, 21, 26, 27, 28, 29, 30, 31, 32, 33, 34, 35, 36]

Eight studies [15, 21, 28, 29, 30, 31, 33, 36] were classified as case series, three [32, 34, 35] as non-RCT and two [26, 27] as before-and-after studies. The sample ranged from nine [21] to 1527 [33] patients. The clinical follow-up assessment after surgery varied considerably, ranging from one week to nine years. Five studies [15, 28, 29, 31, 33] detected the neurosensory dysfunction only by self-reported questionnaires, three [34, 35, 36] did not report the detection method, and the remaining [21, 26, 27, 30, 32] performed physical examinations (thermal and/or sensory) as additional tests. Numbness was the most often reported symptom, followed by paresthesia. Two studies [15, 33] related neuropathies with the distance between the implant and inferior alveolar nerve (IAN), and, despite some dental implants having direct contact with the nerve, all of them showed sensory changes improvement.

**Table 1.** Study characteristics regarding the prevalence of neuropathies after dental implant placement without nerve lateralization (n = 13).

Study characteristics		Sample characteristics			Implant characteristics		Neuropathy characteristics			
Author, year, country	Study design	Quantity (M/F); range age (mean age)	Setting	Analysis time after surgery	Quantity; position	Pre- and post-operative medications	Detection method	Affected sites; symptoms	Reversibility	Conclusion
Abarca *et al*. [29], 2006, Belgium	Case series	58 (45/13); 30 to 71 years (56 years)*	Catholic University Leuven and Erasmus Hospital, Free University of Brussels	8 to 40 months (mean of 20 months)	174*; anterior mandible	NR	Self-administered questionnaire	The gingiva, the inferior lip, and the chin; Numbness, cutting, beating and itching	Yes	33% (n = 19) of patients reported a kind of neurosensory disturbance after the placement of the implants (range 8–24 months)
Bartling, *et al*. [30], 1999, USA	Case series	94 (51/43); NR	Montefiore Medical Center, New York	Post-surgery until 121 days	405; anterior and posterior mandible	NR	Sensitive and pain test with wisp of cotton with swab, soft brush, needle and pointed calliper; thermically test with ice and a mirror handle warmed	NR; Altered sensation (did not specify) and complete anaesthesia	Yes (121 days)	Altered sensation was reported by 1 patient (5.2%) with implants placed in anterior mandible, by 3 patients (7.3%) who had implants placed in posterior mandible, and by 4 patients (11.8%) who had implants placed in both zones
Dannan, *et al*. [28], 2013, Germany	Case series	19 (NR); NR	NR	NR	19; posterior mandible	NR	Self-administered questionnaire	Lower lip, chin, and gingiva; numbness	Both (12 months)	Of these 19 patients, only 5 mentioned the existence of transient (n = 2) or persistent (n = 3) altered sensation
Ellies & Hawker, 1993, Australia	Case series	87 (29/58); (58 years)	Adelaide Dental Hospital and private practice	NR	NR; anterior and posterior mandible	NR	Self-administered questionnaire	The gingiva, the lip, the tongue, and the chin; Numbness, tingling, frozen, pain	Both (6 months)	Altered sensation was reported by 36% of responders, with 23% experiencing transient changes and 13% experiencing persistent changes
Felice *et al*. [34], 2009, Italy	Non-RCT	15 (4/11); 37 to 69 years (56 years)	Different private practices and two hospitals	Recall visits every 4 months until 3 years after prosthetic loading	30 (augmented group) and 26 (short implant); posterior mandible	Pre-operative: 2 g of amoxicillin 1 h prior to procedure; post-operative: 1 g amoxicillin and Ibuprofen 400 mg	NR	Lip and chin, paraesthesia	Yes (3 days)	Two of 15 patients showed transient paraesthesia (13%)
Felice *et al*. [35], 2009, Italy	Non-RCT	Augmentation group: 30 (15/15); 43 to 67 years (55 years); Short implant group: 30 (7/23); 40 to 83 years (56 years)	NR	3 and 10 days; 1, 2, 3 and 4 months	121 (61 of augmentation group and 60 of short implant group); posterior mandible	Pre- and post-operative of augmentation group: 1 g amoxicillin + clavulanic acid and ibuprofen 600 mg	NR	Lip and chin; paraesthesia	Yes (3 days)	In augmentation group, 16 (53%) had transient paraesthesia; and in short implant group, only 2 (6%)
Filipov *et al*. [21], 2023, Romania	Case series	9 (1/8); 58 to 74 years (65.7 ± 5.01 years)	“Queen Maria” Military Hospital, Brasov, Romania	1 and 2 weeks; 2, 6 and 12 months	14; 1 premolar and 13 molars	Pre-operative: 2 g of amoxicillin clavulanate 1 h before surgery Post-operative: amoxicillin clavulanate 1 g/every 12 h and one tablet of Ibuprofen 600 mg at every 8 h	Semmes-Weinstein (SW) pressure neurological test	Lower lip and chin; Hypoesthesia and anesthesia	Both (2 months)	Two patients (22%) showed neurological disturbances one day after surgery. One patient had the nerve recovered in two months and the other had persistent neuropathy during the 3 years of follow-up
Garcia-Blanco, *et al*. [4], 2017, Argentina	Case series	106 (37/69); 25 to 77 years (50 ± 12 years)	Department of dentistry, Buenos Aires University	NR	234; 71 premolars and 163 molars	Post-operative: amoxicillin 500 mg or azithromycin 500 mg and analgesic	NR	NR	Yes (2 months)	Only 1 patient of 106 reported neuropathy due to implant placement
Hartmann, Welte-Jzyk & Seiler, 2017, Germany	Non-RCT	Group A: 20 (10/10); 40 to 73 years (60 years) Group B: 3/5, 30 to 72 years (49 years); Group C: 16/16, 23 to 80 years (58 years)	NR	1 week to 9 years	NR; posterior mandible	Post-operative: amoxicillin or clindamycin	QST	Chin and lower lip; NR	NR	Augmentation procedures did not increase sensory disturbances, indicating no changes in the neurophysiological pathways. None of the patients themselves observed sensory changes after implantation
Porporatti, 2016, Brazil Porporatti *et al*. [27], 2017, Brazil	Before-and-after	20 (6/14); (50.22 ± 6.66 years)	Bauru School of Dentistry, University of São Paulo	1 month and 3 months	NR; anterior and posterior mandible	Post-operative: amoxicillin 500 mg and nimesulide 100 mg	QST	NR; pain and allodynia	NR	There were also no reports of adverse events in the implant placement group
Tejada *et al*. [15], 2022, Brazil	Case series	225 (75/150); (64.1 ± 10 years)	ILAPEO College, Curitiba, Parana	NR	1125; anterior mandible	NR	Questionnaire	NR; pain, tingling and throbbing	Yes (1 to 7 months)	The prevalence of sensory disorders was 4.4% (n = 10)
Vazquez *et al*. [33], 2007, Switzerland	Case series	1527 (637/890); 17 to 86 years (53 years)	University of Geneva, Switzerland	NR	2584; posterior mandible	Pre-operative: antibiotic prophylaxis beginning 1 h before surgery	Self-report	Chin and lower lip; paraesthesia (itching, tingling or prickly sensation)	Yes (3 and 6 weeks without treatment)	There were two cases (0.08% of implants and 0.13% of patients) of postoperative paraesthesia

USA: The United States of America; NR: Not reported; M: male; F: female; RCT: randomized controlled trial; QST: quantitative sensory testing. *data collected through author contact by email.

Neuropathies were reported in 12% (95% CI; 4% to 22%; n = 364) of the patients one week after the implant surgery (Fig. 2). However, regarding studies with three months of follow-up or more, only five studies showed a prevalence different from zero [15, 28, 29, 30, 31] resulting in a prevalence of 5% (95% CI; 1% to 11%; n = 662) (Fig. 3).

**Fig. 2.** Prevalence of neuropathies in one week after dental implant without lateralization. CI: confidence interval.

**Fig. 3.** Prevalence of neuropathies in more than three months after dental implant without lateralization. CI: confidence interval.

#### 3.2.2 Prevalence of neuropathies after implant placement with nerve lateralization (Table 2 and **Supplementary Table 3**) [17, 18, 19, 20, 37, 38, 39, 40, 41, 42, 43, 44, 45, 46, 47, 48, 49, 50, 51, 52]

Twelve studies [18, 19, 20, 42, 43, 44, 45, 46, 47, 48, 49, 51] were classified as case series, seven [37, 38, 39, 40, 41, 50, 52] as before-and-after studies, and one [17] as RCT. The sample size ranged from six [43] to 123 [18] patients. The time between the surgery and the post-operative evaluation varied from one week to ten years. Two [17, 19] studies detected the neurosensory dysfunction only by questionnaire, one [41] did not report the method, and the majority [18, 20, 37, 38, 39, 40, 43, 44, 45, 46, 47, 48, 49, 50, 51, 52] performed a physical examination (thermal and/or sensory) as an additional test. Twenty studies reported the reversibility of neuropathies, and almost half [17, 18, 19, 20, 37, 39, 46, 48, 52] of them were totally reversible, and the longest neuropathy course reported was five years [19]. Hypoesthesia, paresthesia, and numbness were the most frequently reported symptoms. Besides the conventional medicaments, four [20, 39, 50, 52] studies reported other approaches to prevent neuropathies, such as vitamin B complex administration and low-level laser applications.

**Table 2.** Study characteristics regarding the prevalence of neuropathies after dental implant placement with nerve lateralization (n = 20).

Study characteristics		Sample characteristics			Implant characteristics		Neuropathy characteristics			
Author, year, country	Study design	Quantity (M/F); range age (mean age)	Setting	Analysis time after surgery	Implant quantity; lateralization quantity (uni or bilateral)	Pre- and post-operative medications	Detection method	Affected sites; symptoms	Reversibility	Conclusion
Al-Almaie *et al*. [20], 2020, Saudi Arabia	Case Series	8 (3/5); 38 to 57 years (±48 years)	NR	For 2 weeks and for 1, 2, 3, 6, 12, 18 and 24 months postoperatively during the first 2 years and annually thereafter	20; 6 unilaterally and 2 both sides	Post-operative: oral antibiotics, anti-inflammatory nimesulide, analgesic dipyrone, and Vitamin B complex, low-power laser applications	Self-report questionnaire and three tests (light touch, pain and two-point discrimination)	Lower lip and chin; NR	Yes (12 months)	After the 10 IAN transpositions, four patients experienced sensory recovery immediately from local anaesthesia. Six patients had neurosensory disturbance
Atef & Mounir, 2018, Egypt	Before-and-after	7 (7/0); 32 to 53 years (NR)	NR	Weekly basis for the first month, then at months 2, 4 and 6 after surgery	NR; NR	NR	Light touch test, heat test, pain test, and 2-points tactile discrimination test	NR; NR	Yes (3 weeks)	Only 1 patient showed immediate sensation recovery after the resolution of the local anaesthetic effect
Castellano-Navarro *et al*. [18], 2019, Spain	Case Series	123 (33/90); 44 to 68 years (55 years)	NR	24 hours, 1 month, 6 months, and 1 year	337; 107 unilaterally and 16 both sides	NR	Gently pressing the skin and lips with the tip of a probe	Skin and lips; NR	Yes (12 months)	All patients recovered completely, although at different times after the intervention
de Campos *et al*. [17], 2019, Brazil	RCT	Bone graft group: 19; NR (48.55 ± 13.95 years); Control group: 15; NR (51.33 ± 6.71 years)	São Leopoldo Mandic Institute and Research Centre	12 months	47 (bone graft group) and 35 (control group); NR	Pre- and post-operative: amoxicillin, nimesulide, dexamethasone, midazolam, and dipyrone or tylex, depending on the pain	Self-report questionnaire	NR; paraesthesia	Yes (12 months)	All patients reported initial neurosensory disturbance. In the control group, the mean time to recover from sensory disturbances was 118.6 ± 70.13 days, compared with 123.5 ± 140.68 days in bone graft group
Deryabin & Grybauskas, 2021, The USA	Case Series	15 (3/12); 19 to 68 years (NR)	Two centres	10 years (mean: 5.1 years)	48; NR	NR	Self-report questionnaire	Lips; numbness, hypoesthesia, and anaesthesia	Yes (5 years)	All patients reported transient numbness during the first 2 weeks after surgery
Díaz & Gías, 2013, Spain	Before-and-after	15 (14/1); 30 to 64 years (NR)	La Princesa University Hospital department	Third and eighth weeks, and at 6, 12 and 24 months, during the 2 years	38; 11 unilaterally and 4 both sides	Post-operative: amoxicillin-clavulanic acid and ketoprofen	Self-report and a two point-discrimination test	NR; Numbness, tickling sensation and hypoesthesia	Both	8 weeks after surgery, 14 patients had no neurosensory disturbance
Di Pillo & Rapoport, 2009, Brazil	Before-and-after	12 (0/12); 36 to 66 years (48 years)	Ipeno Institute of Florianópolis, Santa Catarina	10 months	28; 8 unilaterally and 2 both sides	Pre-operative: antibiotic therapy together with Diprospan®; Post-operative: Antibiotic, anti-inflammatory, analgesic, Citoneurim® and laser therapy	Small stimuli are performed in the site close to the surgery	NR; paraesthesia	Yes (10 months)	In 12 patients, only one did not have paraesthesia, and the longest healing time was 10 months
Ferrigno, Laureti & Fanali, 2005, Italy	Before-and-after	15 (6/9); 49 to 68 years (58.1 years)	NR	For 2 weeks and for 1, 2, 3, 6, 12, 18 and 24 months postoperatively during the first 2 years, and annually thereafter	46; 11 unilaterally and 4 both sides	Post-operative: oral antibiotics and nonsteroidal analgesics	Self-report questionnaire and three tests (light touch, pain and two-point discrimination)	Lower lip and chin; anaesthesia or burning paraesthesia	Both	Ten patients had neurosensory disturbance. In 6 cases, the patients experienced a total return of sensation within 1 month
Friberg, Ivanoff & Lekholm, 1992, Sweden	Before-and-after	7 (1/6); 41 to 82 years (60 years)	Brânemark Clinic	4 to 16 months (mean follow-up time 10 months)	23; 4 unilaterally and 3 both sides	NR	NR	NR; hypoesthesia and paraesthesia	Both	In one-week follow-up, all patients were with neurosensory disturbance. In 6-months, one patient was with hypoesthesia and one with paraesthesia
Hashemi 2010, Iran	Case Series	87 (47/40); 28 to 54 years (39.3 years)	Implant Department of Tehran University	1 year	NR; 64 unilateral and 23 both sides	NR	Self-report questionnaire	NR; Anaesthesia, hypoesthesia, burning, pain, pinching and tickling	Both	The patients reported neurosensory disturbance in the first week after the operation. The mean duration of them was 37 ± 15 days
Hori *et al*. [43], 2001, Japan	Case Series	6 (3/3); 20 to 61 years (NR)	NR	2.5 years	17; 4 unilateral and 2 both sides	NR	Neurosensory tests (cotton-touch technique and pin-prick test)	Lower lip and skin of the mental area; hypoalgesia, analgesia, hyperalgesia, hypoesthesia, anaesthesia, and hyperesthesia	Both	All the 6 cases displayed anaesthesia and analgesia in both the lower lip and mental skin areas
Kan *et al*. [44], 1997, USA	Case Series	15 (4/11); 48 to 77 years (64 years)	Centre for Prosthodontics and Implant Dentistry at Loma Linda University	10 to 67 months (mean follow-up time 41.3 months)	64; 9 unilateral and 6 both sides	NR	Self-reported questionnaire and neurosensory tests (light touch, brush stroke direction, and two-point discrimination)	Lip and chin; Anaesthesia, paraesthesia, hypoesthesia, tingling, and/or a burning sensation.	Both	The combined total neurosensory disturbance evaluated by light touch, brush stroke direction and two-point tests of the two techniques was 52.4% (11/21)
Khojasteh *et al*. [45], 2016, Iran	Case Series	14 (5/9); 44 to 64 years (53.93 years)	NR	3, 6 and 12 months	51; 5 unilateral and 9 both sides	Pre-operative: amoxicillin or clindamycin, ibuprofen, and dexamethasone. Post-operative: amoxicillin or clindamycin and ibuprofen	Self-report questionnaire, subjective two-point discrimination test, and static light touch test	Lower lip and chin; numbness and tingling	Both	At 12 months, two patients reported numbness, while the remaining patients had regained normal sensation
Lorean *et al*. [46], 2013, Israel	Case Series	57 (11/46); (47.38 ± 14.26 years)	Four centres (1999 to 2009)	once a week for 1 month, then every 2–3 weeks until a full recovery was achieved. The mean follow-up was 20.62 months	232; NR	NR	Two-point discrimination test and pin prick with a sharp instrument.	NR; NR	Yes (6 months)	Four patients reported prolonged transient neural disturbances immediately following surgery (5%). The duration of neural disturbances ranged from 1 to 6 months
Martínez-Rodríguez *et al*. [47], 2016, Spain	Case Series	27 (10/17); 30 to 70 years (57.74 years)	Buccal surgery and implant dentistry service of the study hospital in Madrid	1 week; 3, 6, 12 and 18 months	74; 27 unilaterally	Post-operative: amoxicillin, or clindamycin, and diclofenac sodium	Two-point discrimination test	NR; hypoesthesia	Both	At 3 months postoperative, recovery had reached 74.1%, at 6 months it had reached 88.9%, and at 12 months 92.6% of patients had recovered sensitivity
Mavriqi, Mortellaro & Scarano, 2016, Italy	Case Series	10 (7/3); 40 to 60 years (NR)	NR	Weekly and 1, 2, 3, 6, 12, 24 and 36 months	24; 8 unilateral and 2 both sides	Post-operative: Antibiotic therapy and dexamethasone	Self-report questionnaire, subjective two-point discrimination test, and static light touch test	Lower lip and chin; NR	Yes (3 months)	In 10 of the 12 surgical sites, the function of the IAN restored in 2 weeks
Morrison, Chiarot & Kirby, 2002, Canada	Case Series	12 (NR); NR	Queen Elizabeth II Health Sciences Centre in Halifax, Nova Scotia	6 to 60 months (mean follow-up time 16 months)	30; 4 unilateral and 8 both sides	NR	Self-report questionnaire, two-point discrimination test, brush-stroke directional discrimination, sharp/dull discrimination, and static light touch test	Lip and chin; numbness, dysesthesia, pain, and tingling	Both	80% of the sites had returned to normal. Four patients (4 sites in total) reported that the change in sensation was persistent
Nishimaki *et al*. [50], 2016, Japan	Before-and-after	7 (1/6); 38 to 75 years (64 years)	Shinshu University School of Medicine	12 and 105 months (mean follow-up time 49 months)	22; 6 unilateral and 1 both sides	Post-operative: dexamethasone and six patients received oral vitamin B12	Modified Semmes-Weinstein perception test	Lower lip and chin; hypoesthesia	Both	Complete recovery of neural function was observed on two sides
Peleg *et al*. [51], 2002, Israel	Case Series	10 (2/10); 47 to 67 years (56 ± 7 years)	NR	16 to 46 months (mean follow-up time 29.8 months ±10)	23; NR	NR	Pin-prick sensation test	NR; Hypoesthesia and paraesthesia	Both	Four patients experienced sensory recovery immediately after the local anaesthesia. Six patients had hypoesthesia immediately after the procedure
Rathod *et al*. [52], 2019, India	Before-and-after	10 (NR); NR	Bharati Vidyapeeth University and Dental College and Hospital, Pune	1st and 7th days and every month	NR; NR	Post-operative: Methylcobalamin	Semmes-Weinstein monofilaments	NR; NR	Yes (4 months)	The minimum time required for complete recovery was 2.0 months, and maximum was 4.0 months.

*IAN: inferior alveolar nerve; RCT: randomized controlled trial; NR: not reported; M: male; F: female.*

Nine studies [17, 19, 38, 41, 42, 43, 47, 50, 52] showed 100% of neuropathies one-week post-surgery, and the overall prevalence was 90% (95% CI; 72% to 100%; n = 503) (Fig. 4). Three months or more after surgery, two studies [37, 51] showed no occurrence (0%) of neuropathies, and the overall prevalence for this follow-up time was 42% (95% CI; 24% to 60%; n = 493) (Fig. 5).

**Fig. 4.** Prevalence of neuropathies in one week after dental implant with lateralization. CI: confidence interval.

**Fig. 5.** Prevalence of neuropathies in more than three months after dental implant with lateralization. CI: confidence interval.

#### 3.2.3 Management of neuropathies associated with dental implant placements (Table 3) [53, 54, 55, 56, 57]

Three [54, 55, 56] were classified as before-and-after studies, one [57] was a RCT, and the other [53] was a non-RCT. Five studies evaluated different management approaches for neuropathies caused by dental implant placement. The sample size varied between 16 [56] and 64 [54] patients and the follow-up from seven days [56] to seven years [54]. More than half of the studies [53, 56, 57] diagnosed the neuropathies based on clinical evaluation, and the other two [54, 55] according to patient self-report. The IAN was the most affected nerve, and the lip, chin and tongue were the most affected sites. The five studies reported pharmacological treatment; however, two compared it to another management alternative.

**Table 3.** Study characteristics regarding management of neuropathies associated with dental implant placement (n = 5).

Study characteristics		Sample characteristics			Neuropathy characteristics				
Author, year, country	Study design	Quantity (M/F); range age (mean age)	Setting	Analysis time after implant surgery and treatment	Detection method	Affected sites; symptoms	Nerve affected; reversibility	Treatment	Conclusion
Gagik *et al*. [53], 2020, Armenia	Non-RCT	27 (11/16); NR (41.7 years)	NR	NR	Neurosensory testing (needle puncture and thermal - hot water tube) and visual analogue scale	Lips and chin; paraesthesia or hyperesthesia	IAN; NR	Anti-inflammatory, analgesics, antioxidants, B complex of the vitamins group. For internal use, neurorubine (B1, B6, B12) is prescribed once a day for 3 weeks, ibuprofen 600 mg three times a day for 3 weeks, oral dexamethasone 4 mg 2 tablets for 3 days and 1 tablet for next 3 days, in case of pain, Ketonal Forte 100 mg, 1–2 tablets per day. The Milta-F-8-01 device includes low-intensity pulse lasers, a magnetic field generator, low-intensity laser radiation, and a combined physiotherapeutic effect of the magnetic field. Pulsed wave frequency 80 Hz, wavelength 0.89 μm, radiation power 1.2–5 mW/cm^2^, magnetic field is 5–10 mTl, for 5 minutes. Magnetic-laser therapy was carried out for 10–14 days, intraoral and extraoral method in the projection of the inferior alveolar nerve and mental foramen	Magnetic-laser therapy can have a positive effect on the restoration of disorders of the sensitivity of the lower alveolar nerve, accelerating the improvement of the regeneration of the affected nerves after dental implantation, increases the effectiveness of treatment
Ghasemi *et al*. [57], 2022, Iran	RCT	Control group: 23 (10/13) Case group: 23 (9/14)	Tabriz University of Medical Sciences, Iran	4 days and 1, 2 and 3 months	Neurosensory examination Tests and 10-cm visual analogy scale	NR; paraesthesia and pain	IAN; NR	Pre- and post-operative: amoxicillin and ibuprofen 600 mg Case group: daily vitamin B complex (5 mg of B1; 2 mg of B2 and B6; 20 mg of nicotinamide) starting immediately after surgery	There was no significant difference between the mean pain intensity and paraesthesia in the intervention and control groups at all the follow-up intervals
Juodzbalys *et al*. [59], 2011, Lithuania	Before-and-after	16 (8/8); 36 to 65 years (52.2 ± 8.1 years)	Lithuanian University of Health Sciences, Kaunas, Lithuania	10–52 h; 7, 14 and 21 days, 1, 2 and 3 months	Self-report and clinical symptoms (comparation of pain detection threshold at the skin of innervation zone of the healthy and affected sides)	NR; hyperalgesia and hypoalgesia	IAN; both	Six-step IAN injury during dental implant surgery (IANIDIS) protocol: Removal of the implant, within 36 h post-surgery. Subsequently, any irritants (bone debris, hematoma) in close approximation were removed. If during surgery, known or observed trauma (including traction or compression of the nerve trunk) has occurred, the topical application of intravenous form steroids, one to two millilitres of dexamethasone (4 mg/mL), was applied for 1–2 min.	A six-step protocol aimed at managing patients with IAN injury during dental implant surgery was a useful tool that could provide successful treatment outcome in mild and moderate cases
Kim *et al*. [54], 2013, South Korea	Before-and-after	64 (29/35); NR	Seoul National University Dental Hospital	10.91 months (from 1 week to 5 years); 30.18 (from 2 months to 7 years and 7 months)	Self-assessment of the neurosensory function in terms of reduced function and neurogenic discomfort	Lip, chin, and tongue; hypoesthesia, anaesthesia, paraesthesia, dysesthesia	IAN; NR	Month 0: Prednisolone 5 mg 7 days and Neurontin 300 mg, then 600–800 mg. Months 1 and 2: Vitamin B12, 1, 6 (Beecom 1 T), Aspirin 1 T, Ginkgo-biloba (Ginkomin 1 T), Amitriptyline 10 mg, 20 mg, 30 mg, 40 mg thereafter Prn) and Tramadol 150 mg. Months 4–8: B12, 1, 6, nonsteroidal anti-inflammatory drugs, Ginkomin and Tramadol. In all months, hot pack, massage, laser, electrical acupuncture stimulation therapy, stellate ganglion block.	Groups I (first visit time to our department after nerve damage <9 months) and III (implant removal or decompression before the study) exhibited greater improvement in symptoms than groups II (first visit time to our department after nerve damage >9 months) and IV (no treatment or medication before the study), but the difference was not statistically significant
Park, Lee, and Kim, 2010, South Korea	Before-and-after	47 (15/32); NR (47.7 years)	Yonsei University Dental Hospital	From less than 3 months to more than 12 months; 3 months	10-point visual analogy scale and were questioned on the factors affecting pain relief	NR; pain or abnormal sensation	Trigeminal nerve; NR	It was initially prescribed 300 mg of gabapentin and the dosage was gradually titrated up to 1800 to 2400 mg for 1 month and re-evaluated. Patients who developed side effects or reported inefficacy with gabapentin were prescribed a tricyclic antidepressant such as nortriptyline or amitriptyline (10 to 75 mg), topiramate (25 to 100 mg), and venlafaxine XR (37.5 to 75 mg) for the next 2 months	Patients started with pharmacotherapy early after nerve injuries showed better results. Patients treated with anticonvulsants and antidepressants within the first 3 months showed the maximum reduction in pain

*IAN: inferior alveolar nerve; RCT: randomized controlled trial; NR: not reported; M: male; F: female.*

##### 3.2.3.1 Only pharmacological treatments (analgesic, anti-inflammatory, anticonvulsant and/or antidepressant)

Juodzbalys *et al*. [56], 2011: The management was conducted according to the severity of neuropathy (hyperalgesia and/or hypoalgesia), and it was assessed using the asymmetry score, according to Sakavicius *et al*. [58] 2008. In cases with a mild degree, 400–600 mg ibuprofen three times daily for one week was prescribed. In patients of moderate or severe degree, dexamethasone 4 mg, two tablets for three days and one tablet for the next three days or oral prednisolone 1 mg per kg per day was prescribed. Ibuprofen 800 mg was considered an alternative or adjunct medication. Adding to that, diuretics, vasodilators, and B group vitamins and antihistaminic drugs were prescribed in all groups. They monitored the patients after 7, 14 and 21 days, 1, 2 and 3 months. It was concluded that this protocol provided successful treatment outcomes in mild and moderate cases.

Kim *et al.* [54], 2013: Different management was conducted to treat hypoesthesia, anesthesia, paresthesia and/or dysesthesia for each month of treatment. In the first month, the patients took prednisolone (5 mg, three times per day for seven days) and gabapentin 300 mg, then 600–800 mg (three times per day); in the second and third months, they took vitamin B12, 1, 6 (one tablet three times per day), nonsteroidal anti-inflammatory drugs (acetylsalicylic acid one tablet three times per day), ginkgo-biloba (Ginkomin® one tablet three times per day), tricyclic antidepressant (Amitriptyline 10 mg, 20 mg, 30 mg and 40 mg), and if necessary, tramadol 150 mg; in months four to eight, they took B12, 1, 6, nonsteroidal anti-inflammatory drugs, ginkgo-biloba, gabapentin plus tricyclic antidepressants, and, if necessary, tramadol again. In addition, hot packs, massages, laser therapy, electrical acupuncture stimulation therapy, and stellate ganglion blocks were done during the whole period. The authors concluded that, in the overall sample, nine (16%) patients had improved tactile sensations. Patients who visited the clinic within nine months of nerve injury (24%) and whose implants were surgically decompressed (23%) exhibited more remarkable improvement in symptoms than patients who visited the clinic nine months after nerve injury (5%) and who had not undergone any treatment or medication (12%), but the difference was not statistically significant.

Park, Lee and Kim, 2010 [55]: To treat pain and/or abnormal sensation, it was initially prescribed 300 mg of gabapentin and the dosage was gradually titrated up to 1800 to 2400 mg for one month and reevaluated. Patients who developed side effects or reported inefficacy with gabapentin were prescribed a tricyclic antidepressant such as nortriptyline or amitriptyline (10 to 75 mg), topiramate (25 to 100 mg), and venlafaxine XR (37.5 to 75 mg) for the next two months. Patients who started pharmacotherapy within three months after nerve injury and the between 3 to 6 months showed a 37% and 27.1% decrease in pain on the visual analog scale, respectively. The group prescribed medication 6 to 12 months and more than 12 months after nerve injury showed a 22.2% and 17.1% reduction in pain, respectively. The group taking gabapentin reported a 45.8% reduction in total pain, while the gabapentin and the tricyclic antidepressant group reported a 22.2% decrease.

##### 3.2.3.2 Low-level laser therapy [53]

The sample was divided into two groups receiving the same drugs. The difference was the administration of low-intensity laser radiation (Milta-F-8-01) in one group. The pharmacological protocol to treat paresthesia and/or hyperesthesia included ibuprofen 600 mg (three times a day for three weeks) and dexamethasone 4 mg (two tablets for three days), Ketonal Forte® 100 mg (one or two tablets per day), antioxidants and B complex vitamins (Neurorubine® B1, B6 and B12 once a day for three weeks). Magnetic-laser therapy (pulsed wave frequency 80 Hz, wavelength 0.89 μm, radiation power 1.2–5 mW/cm^2^, the magnetic field was 5–10 mTl) was carried out for five minutes, 10–14 days, the intraoral and extraoral method, in the projection of the IAN and mental foramen. It was concluded that low-intensity laser radiation could positively affect the reverse disorders of the lower alveolar nerve sensitivity, accelerating the improvement of the regeneration of the affected nerves after dental implantation.

##### 3.2.3.3 Vitamin B complex [57]

Although all patients were diagnosed with some neuropathy (paraesthesia and/or pain), the sample was divided into intervention and control groups. All patients received ibuprofen 600 mg and chlorohexidine mouthwash. The intervention group received daily vitamin B complex tablets, including 5 mg of vitamin B1, 2 mg of vitamins B2 and B6, and 20 mg of nicotinamide, starting immediately after implantation. Standard neurosensory examination tests evaluated changes in sensation in the field. The patients were monitored at intervals of 14 days and 1, 2 and 3 months after treatment. The rate of paresthesia in the control group did not differ significantly from the intervention group.

### 3.3 Methodological quality in studies

#### 3.3.1 Case series (**Supplementary Table 4A**)

Ten [15, 21, 28, 29, 30, 31, 33, 45, 47, 49] presented high, seven [19, 20, 36, 42, 44, 46, 48] moderate, and three [18, 43, 51] low methodological quality. Most studies did not mention the sample’s eligibility criteria (first checklist item) or how the authors evaluated and calculated the results (last item). Two JBI tool items did not apply to these studies. One was regarding the follow-up because it was not mandatory for the included studies. The other referred to demographic information because a clear description of sociodemographic variables and geographic regions was not mandatory either.

#### 3.3.2 Before-and-after studies and non-randomized controlled trials (**Supplementary Table 4B**)

None studies from this group presented low methodological quality. Four studies were non-RCT, [32, 34, 35, 53] and just one [32] showed high methodological quality. Among before-and-after studies, six [27, 37, 52, 54, 55, 56] presented high, and five [38, 39, 40, 41, 50] a moderate methodology quality. For before-and-after studies, the fourth question was not applicable because this study design did not have a control group. Two JBI checklist items were responsible for decreasing the methodological quality. The first item was the reliability of neuropathy detection methods, where many studies lacked referencing the sources. The other (item 5) considers the outcome measurement pre- and post-intervention. In other words, whether the authors also performed sensory tests before surgery; however, most studies did not mention this information.

#### 3.3.3 Randomized controlled trial (**Supplementary Table 4C**)

One [57] study was categorized as moderate, and another as low [17] methodological quality. The items that differentiated both studies were the reliability of neuropathy detection (item 11) and the assessors blinding (item 6).

## 4. Discussion

The normal somatic sensation is processed continuously and is an unconscious activity. Meanwhile, a disordered sensation is alarming and dominates the patient’s attention. Due to its neuroanatomical location, neurosensory disturbances regularly occur in the mandible after dental surgeries. Anatomical factors, the handling of the IAN nerve during surgery, or the perforation of the nerve canal can all facilitate such disturbances [29]. This systematic review synthesizes the available evidence that assessed the prevalence of neuropathies and/or nerve injuries after dental implant placement in the mandible and the tested management. Thirty-eight studies were included, and three outcomes were evaluated.

The first outcome reported the prevalence of neuropathies within patients who underwent dental implant placement without lateralization. There was a significant difference between the lowest reported prevalence [33] (0%) and the highest one [35] (60%) at both follow-up times (one week and more than three months). The differences between these two studies might be explained by numerous motives, such as dental implant size and localization, techniques modalities, medications taken before and after surgery and study design. The study with the highest prevalence was a non-RCT assessing two surgical procedures in different evaluation times and control samples. The patient’s knowledge of the study purpose and their assignment group may have caused a Hawthorne effect—overreporting sensations/feelings. Moreover, the case group of this study [35] underwent vertical bone augmentation surgery through a bone block approach after the osteotomy, leaving a distance from the bone crest to the mandibular canal of around 2–4 mm. Conversely, the other study [33] was observational, with a larger sample, no details specifying the surgical complexity, and a self-reported neuropathic evaluation that was not detailed, possibly preventing a memory bias.

Regarding the second outcome (lateralization of the IAN), a higher prevalence of neuropathies was already expected since the dentist directly manipulates the nerve during surgery. Nevertheless, one study [46] showed a prevalence lower than 50% during the first week of post-surgery follow-up. The correct use of piezosurgical instruments might explain this low percentage, as reduced damage to delicate tissues is observed after gentle use of this device. Moreover, choosing the correct technique, avoiding overheating, mental nerve overstretching, and careful nerve dissection can contribute to low rates of neurosensory disturbance [46, 48]. Above three months follow-up, ten [17, 18, 19, 39, 41, 43, 45, 50, 52] of eighteen studies showed a prevalence higher than 50%, and the overall prevalence decreased compared to the first-week post-surgery.

The reason for a higher prevalence of neuropathies linked to dental implant placement in the mandible is the uncertain localization of the IAN and the mandible atrophy. The studies of the second outcome evaluated the nerve’s localization while performing the lateralization; however, not all other included studies mentioned this information. Bartling *et al*. [30] placed dental implants 2 mm and 1 mm above the alveolar canal. The prevalence of neuropathies through their panoramic image sample did not differ from that assessed in the computed tomography sample. Another study [28] evaluated 29 patients whose distance between the implant tip and mandibular canal upper wall was less than 2 mm, and five patients developed neuropathies. Limited evidence exists regarding the proper distance between the implant and the mandibular canal to prevent nerve damage [59, 60]. In this regard, a recent study [61] reported that a distance of 0.75 mm did not damage the nerve.

All five studies mentioned the pharmacological management among the available treatments for neuropathies caused by dental implants. The facility to get medicines, either by the patient or by financing the studies, and being a non-invasive procedure makes this type of management more common. Steroids [17, 39, 45] such as dexamethasone 4 mg and vitamin B complex [20, 39, 50, 52] were prescribed to avoid the possible neuropathy in pre and/or post-surgery studies [17, 39, 45] with IAN lateralization; however, all of them showed a high prevalence of neuropathies, in both follow-up analysis. The efficacy of vitamin B complex in neuropathy prevention agreed to Ghasemi *et al*. [57], according to which the vitamin did not influence nerve recovery. Additionally, one article [62] showed that the level of vitamin B in the blood decreases over days after nerve injury, requiring replacement in some cases. Low-laser therapy was also mentioned [53] as a treatment and showed promising results, accelerating the improvement of the regeneration of the affected nerves. Two studies [20, 39] applied laser after surgery, but only one [20] showed a low prevalence of neuropathies. This data is only partially reliable, as none explained how they applied this laser and how many sections were performed.

The main limitation of this systematic review is regarding the heterogeneity of study design. When dealing with signs and/or symptoms after surgery, the ideal is intervention studies. However, the majority of studies included in this systematic review were observational, due to the ease of implementing the methodology and obtaining a larger sample. In the studies [28, 31] in which the authors contacted patients (by telephone or by email) to ask some data about the post-operative period, there may have been memory bias, since follow-up could have lasted from months to years. Or, if the study authors reported the information found in medical records [15, 33], one cannot be sure that the patient assessment was carried out in a standardized way. This limitation—the authors’ lack of control over patients—must be highlighted as it may interfere with prevalence results. Another limitation, which is a consequence of the one mentioned above, is the lack of information on the characteristics of the implants used and the level of training of surgeons, to see if it is related to the prevalence of neuralgia.

Future studies must standardize the diagnostic criteria and the imaging process and provide more details about patients’ profiles and dental implant placement techniques. Besides that, how digital dentistry treatment planning and navigated implant surgery may contribute to further reducing the chances of neurosensory alterations should be assessed.

## 5. Conclusion

In mandible, implant surgeries without nerve lateralization, 11% of the patients presented neuropathy after one week. However, this prevalence reduced to 5% after three months. In implant surgeries with lateralization of the nerve, the prevalence of neuropathy was higher, from 90% in the first week to 42% after three months.

Pharmacologic treatments and low-laser therapy showed efficacy in the neuropathy’s treatment. In contrast, including vitamin B in pharmacological treatment presented no benefit for neuropathies management.

## 6. Protocol and registration

This systematic review was reported according to the Preferred Reporting Items for Systematic reviews and Meta-Analyses (PRISMA) [63] Checklist. The systematic review protocol was registered in the International Prospective Register of Systematic Review (PROSPERO) platform under number CRD42023449556.

## 7. Highlights

 Neuropathies are detected in 5% to 11% of implants surgeries without nerve lateralization.

Neuropathies are detected in 42% to 90% of implants surgeries with nerve lateralization.

Among many available treatments, the pharmacological has the higher number of scientific publications.

Neuropathies are detected more in jaw than maxilla.

## Supplementary Material


