# Supplementary material for: Prevalence and management of neuropathic injury caused by dental implant insertion in mandible: a systematic review
Source: J Oral Facial Pain Headache. 2024 Jun 12;38(2):25–47. doi: 10.22514/jofph.2024.012 (PMC11810661; doi:10.22514/jofph.2024.012)
Supplement: Supplementary file 1 [file Supplementary-material.docx]

Supplementary material

Supplementary Table 1. Data search strategy.

| Database | Search query  01 December 2023 |
| --- | --- |
| Cochrane Central Register for Controlled Trials | #7 - #3 AND #6  #6 - #4 AND #5  #5 - (“mandibular canal” OR “inferior alveolar nerve” OR “mental nerve” OR “mandibular nerve” OR “maxillary nerve” OR “palatine nerve” OR “infraorbital nerve” OR “facial nerve” OR “trigeminal nerve” OR “mandibular branch” OR “mandibular branches” OR “maxillary branch” OR “maxillary branches” OR “maxillary branch”):ti,ab,kw  #4 - (“disturbance” OR “disturbances” OR “pain” OR “injury” OR “injuries” OR “injured” OR “damage” OR “damages” OR “hurt” OR “altered sensation” OR “sensory alterations” OR “sensory disturbance” OR “sensory disturbances” OR “neurosensory disturbance” OR “neurosensory disturbances” OR “allodynia” OR “hyperalgesia” OR “paresthesia” OR “dysesthesia” OR “hypoesthesia” OR “hypesthesia” OR “numbness” OR “neuropathy” OR “neuropathies” OR “neuropathic” OR “painful post-traumatic trigeminal neuropathy” OR “Neuropathic Pain” OR “Atypical Neuralgia” OR “Atypical Neuralgias” OR “Mandibular Nerve Injuries” OR “Trigeminal Nerve Diseases” OR “Cranial Nerve Injuries” OR “Trigeminal Nerve Injuries” OR “trigeminal neuralgia” OR “Facial Pain” OR “Facial Neuralgia” OR “Facial Nerve Injuries” OR “Glossopharyngeal Nerve Diseases” OR “idiopathic pain”):ti,ab,kw  #3 - #1 OR #2  #2 - (“Dental Implants” OR “Dental Implantation” OR “Dental Implant”):ti,ab,kw  #1 - (implant OR implants):ti,ab,kw AND (oral OR teeth OR tooth OR incisor OR canine OR premolar OR molar OR mandible OR maxilla OR jaw):ti,ab,kw |
| Embase | ((implant:ab,ti OR implants:ab,ti) AND (oral:ab,ti OR teeth:ab,ti OR tooth:ab,ti OR incisor:ab,ti OR canine:ab,ti OR premolar:ab,ti OR molar:ab,ti OR mandible:ab,ti OR maxilla:ab,ti OR jaw:ab,ti) OR ‘dental implants’:ab,ti OR ‘dental implantation’:ab,ti OR ‘dental implant’:ab,ti) AND (‘disturbance’:ab,ti OR ‘disturbances’:ab,ti OR ‘pain’:ab,ti OR ‘injury’:ab,ti OR ‘injuries’:ab,ti OR ‘injured’:ab,ti OR ‘damage’:ab,ti OR ‘damages’:ab,ti OR ‘hurt’:ab,ti OR ‘altered sensation’:ab,ti OR ‘sensory alterations’:ab,ti OR ‘sensory disturbance’:ab,ti OR ‘sensory disturbances’:ab,ti OR ‘neurosensory disturbance’:ab,ti OR ‘neurosensory disturbances’:ab,ti OR ‘allodynia’:ab,ti OR ‘hyperalgesia’:ab,ti OR ‘paresthesia’:ab,ti OR ‘dysesthesia’:ab,ti OR ‘hypoesthesia’:ab,ti OR ‘hypesthesia’:ab,ti OR ‘numbness’:ab,ti OR ‘neuropathy’:ab,ti OR ‘neuropathies’:ab,ti OR ‘neuropathic’:ab,ti OR ‘painful post-traumatic trigeminal neuropathy’:ab,ti OR ‘neuropathic pain’:ab,ti OR ‘atypical neuralgia’:ab,ti OR ‘atypical neuralgias’:ab,ti OR ‘mandibular nerve injuries’:ab,ti OR ‘trigeminal nerve diseases’:ab,ti OR ‘cranial nerve injuries’:ab,ti OR ‘trigeminal nerve injuries’:ab,ti OR ‘trigeminal neuralgia’:ab,ti OR ‘facial pain’:ab,ti OR ‘facial neuralgia’:ab,ti OR ‘facial nerve injuries’:ab,ti OR ‘glossopharyngeal nerve diseases’:ab,ti OR ‘idiopathic pain’:ab,ti) AND (‘mandibular canal’:ab,ti OR ‘inferior alveolar nerve’:ab,ti OR ‘mental nerve’:ab,ti OR ‘mandibular nerve’:ab,ti OR ‘maxillary nerve’:ab,ti OR ‘palatine nerve’:ab,ti OR ‘infraorbital nerve’:ab,ti OR ‘facial nerve’:ab,ti OR ‘trigeminal nerve’:ab,ti OR ‘mandibular branch’:ab,ti OR ‘mandibular branches’:ab,ti OR ‘maxillary branches’:ab,ti OR ‘maxillary branch’:ab,ti) AND [embase]/lim NOT ([embase]/lim AND [medline]/lim) |
| LILACS | (((implant OR implants OR implante OR implantes) AND (oral OR teeth OR tooth OR incisor OR canine OR premolar OR molar OR mandible OR maxilla OR jaw OR oral OR orais OR dentes OR dientes OR dente OR diente OR dentales OR incisivo OR canino OR pré-molar OR premolar OR molar OR mandíbula OR maxila OR maxilar))) AND (((disturbance OR disturbances OR pain OR injury OR injuries OR injured OR damage OR damages OR hurt OR “altered sensation” OR “sensory alterations” OR “sensory disturbance” OR “sensory disturbances” OR “neurosensory disturbance” OR “neurosensory disturbances” OR allodynia OR hyperalgesia OR paresthesia OR dysesthesia OR hypoesthesia OR hypesthesia OR numbness) OR (neuropathy OR neuropathies OR neuropathic OR “painful post-traumatic trigeminal neuropathy” OR “Neuropathic Pain” OR “Atypical Neuralgia” OR “Atypical Neuralgias” OR “Mandibular Nerve Injuries” OR “Trigeminal Nerve Diseases” OR “Cranial Nerve Injuries” OR “Trigeminal Nerve Injuries” OR “trigeminal neuralgia” OR “Facial Pain” OR “Facial Neuralgia” OR “Facial Nerve Injuries” OR “Glossopharyngeal Nerve Diseases” OR “idiopathic pain” OR distúrbio OR distúrbios OR disurbios OR perturbación OR dor OR dolor OR injúria OR injúrias OR lesión OR lesiones OR dano OR danos OR daño OR “sensação alterada” OR “sensações alteradas” OR “alteração sensorial” OR “alterações sensoriais” OR “alteración sensorial” OR “alteraciones sensoriales” OR “cambios sensoriales” OR “distúrbio sensorial” OR “distúrbios sensoriais” OR “distúrbios neurossensoriais” OR “trastornos neurosensoriales” OR “trastorno neurossensorial” OR alodínia OR alodinia OR hiperalgesia OR parestesia OR disestesia OR hipoestesia OR dormência OR entumecimiento OR neuropatia OR neuropatía OR neuropatias OR neuropatías OR neuropática OR neuropático OR “Neuralgia atípica” OR “neuralgias atípicas” OR “injúrias do nervo mandibular” OR “lesiones del nervio mandibular” OR “injúria do nervo mandibular” OR “lesión del nervio mandibular” OR “doença do nervo trigêmeo” OR “enfermedad del nervio trigémino” OR “doenças do nervo trigêmeo” OR “enfermedades del nervio trigémino” OR “doenças do nervo trigeminal” OR “doença do nervo trigemial” OR “injúria do nervo trigeminal” OR “injúrias do nervo trigeminal” OR “injúria do nervo trigêmeo” OR “injúrias do nervo trigêmeo” OR “neuralgia do trigêmeo” OR “neuralgias do trigêmeo” OR “neuralgia trigeminal” OR “neuralgias trigeminais” OR “neuralgia trigeminal” OR “dor facial” OR “dolor facial” OR “dores faciais” OR “neuralgia facial” OR “neuralgias faciais” OR “injúria do nervo facial” OR “lesión del nervio facial” OR “injúrias do nervo facial” OR “lesiones del nervio facial” OR “dor idiopática” OR “dolor idiopático”))) AND ( db:(“LILACS”)) |
| Medline/PubMed | #3 - #1 AND #2  #2 - ((“mandibular canal”[Title/Abstract] OR “inferior alveolar nerve”[Title/Abstract] OR “mandibular nerve”[MeSH Terms] OR “mental nerve”[Title/Abstract] OR “mandibular nerve”[Title/Abstract] OR “maxillary nerve”[Title/Abstract] OR “maxillary nerve”[MeSH Terms] OR “palatine nerve”[Title/Abstract] OR “infraorbital nerve”[Title/Abstract] OR “facial nerve”[MeSH Terms] OR “facial nerve”[Title/Abstract] OR “trigeminal nerve”[MeSH Terms] OR “trigeminal nerve”[Title/Abstract] OR “mandibular branch”[Title/Abstract] OR “mandibular branches”[Title/Abstract] OR “maxillary branch”[Title/Abstract] OR “maxillary branches”[Title/Abstract] OR ((“mandibular”[Title/Abstract] OR “jaw”[Title/Abstract] OR “jaw”[MeSH Terms] OR “mandible”[Title/Abstract] OR “mandible”[MeSH Terms] OR “maxilla”[Title/Abstract] OR “maxilla”[MeSH Terms]) AND (“nerve”[Title/Abstract] OR “nerves”[Title/Abstract] OR “branch”[Title/Abstract] OR “branches”[Title/Abstract] OR “canal”[Title/Abstract]))) AND (“disturbance”[Title] OR “disturbances”[Title] OR “pain”[Title] OR “injury”[Title] OR “injuries”[Title] OR “injured”[Title] OR “damage”[Title] OR “damages”[Title] OR “hurt”[Title] OR “altered sensation”[Title] OR “sensory alterations”[Title] OR “sensory disturbance”[Title] OR “sensory disturbances”[Title] OR “neurosensory disturbance”[Title] OR “neurosensory disturbances”[Title] OR “allodynia”[Title] OR “hyperalgesia”[Title] OR “hyperalgesia”[MeSH Terms] OR “paresthesia”[Title] OR “paresthesia”[MeSH Terms] OR “dysesthesia”[Title] OR “hypoesthesia”[Title] OR “hypesthesia”[MeSH Terms] OR “numbness”[Title])) OR (“neuropathy”[Title/Abstract] OR “neuropathies”[Title/Abstract] OR “neuropathic”[Title/Abstract] OR “painful post-traumatic trigeminal neuropathy”[Title/Abstract] OR “Neuropathic Pain”[Title/Abstract] OR “Atypical Neuralgia”[Title/Abstract] OR “Atypical Neuralgias”[Title/Abstract] OR “Mandibular Nerve Injuries”[MeSH Terms] OR “Mandibular Nerve Injuries”[Title/Abstract] OR “Trigeminal Nerve Diseases”[MeSH Terms] OR “Trigeminal Nerve Diseases”[Title/Abstract] OR “Cranial Nerve Injuries”[MeSH Terms] OR “Cranial Nerve Injuries”[Title/Abstract] OR “Trigeminal Nerve Injuries”[MeSH Terms] OR “Trigeminal Nerve Injuries”[Title/Abstract] OR “trigeminal neuralgia”[MeSH Terms] OR “trigeminal neuralgia”[Title/Abstract] OR “Facial Pain”[MeSH Terms] OR “Facial Pain”[Title/Abstract] OR “Facial Neuralgia”[MeSH Terms] OR “Facial Neuralgia”[Title/Abstract] OR “Facial Nerve Injuries”[MeSH Terms] OR “Facial Nerve Injuries”[Title/Abstract] OR “Glossopharyngeal Nerve Diseases”[MeSH Terms] OR “Glossopharyngeal Nerve Diseases”[Title/Abstract] OR “idiopathic pain”[Title/Abstract])  #1 - ((“implant”[Title/Abstract] OR “implants”[Title/Abstract]) AND (“dent*”[Title/Abstract] OR “oral”[Title/Abstract] OR “teeth”[Title/Abstract] OR “tooth”[Title/Abstract] OR “prosthes*”[Title/Abstract] OR “incisor”[Title/Abstract] OR “canine”[Title/Abstract] OR “premolar”[Title/Abstract] OR “molar”[Title/Abstract] OR “mandible”[Title/Abstract] OR “maxilla”[Title/Abstract] OR “jaw”[Title/Abstract])) OR (“Dental Implants”[MeSH Terms] OR “Dental Implants”[Title/Abstract] OR “Dental Implantation”[Title/Abstract] OR “Dental Implant”[Title/Abstract]) |
| SCOPUS | (TITLE-ABS-KEY(implant OR implants) AND TITLE-ABS-KEY(oral OR teeth OR tooth OR incisor OR canine OR premolar OR molar OR mandible OR maxilla OR jaw) OR (“Dental Implants” OR “Dental Implantation” OR “Dental Implant”)) AND (TITLE-ABS-KEY(“disturbance” OR “disturbances” OR “pain” OR “injury” OR “injuries” OR “injured” OR “damage” OR “damages” OR “hurt” OR “altered sensation” OR “sensory alterations” OR “sensory disturbance” OR “sensory disturbances” OR “neurosensory disturbance” OR “neurosensory disturbances” OR “allodynia” OR “hyperalgesia” OR “paresthesia” OR “dysesthesia” OR “hypoesthesia” OR “hypesthesia” OR “numbness” OR “neuropathy” OR “neuropathies” OR “neuropathic” OR “painful post-traumatic trigeminal neuropathy” OR “Neuropathic Pain” OR “Atypical Neuralgia” OR “Atypical Neuralgias” OR “Mandibular Nerve Injuries” OR “Trigeminal Nerve Diseases” OR “Cranial Nerve Injuries” OR “Trigeminal Nerve Injuries” OR “trigeminal neuralgia” OR “Facial Pain” OR “Facial Neuralgia” OR “Facial Nerve Injuries” OR “Glossopharyngeal Nerve Diseases” OR “idiopathic pain”)) AND (TITLE-ABS-KEY(“mandibular canal” OR “inferior alveolar nerve” OR “mental nerve” OR “mandibular nerve” OR “maxillary nerve” OR “palatine nerve” OR “infraorbital nerve” OR “facial nerve” OR “trigeminal nerve” OR “mandibular branch” OR “mandibular branches” OR “maxillary branch” OR “maxillary branches” OR “maxillary branch”)) |
| Web of Science | (((TS=(implant OR implants)) AND TS=(oral OR teeth OR tooth OR incisor OR canine OR premolar OR molar OR mandible OR maxilla OR jaw)) OR TS=(“Dental Implants” OR “Dental Implantation” OR “Dental Implant”)) AND ((TS=(“disturbance” OR “disturbances” OR “pain” OR “injury” OR “injuries” OR “injured” OR “damage” OR “damages” OR “hurt” OR “altered sensation” OR “sensory alterations” OR “sensory disturbance” OR “sensory disturbances” OR “neurosensory disturbance” OR “neurosensory disturbances” OR “allodynia” OR “hyperalgesia” OR “paresthesia” OR “dysesthesia” OR “hypoesthesia” OR “hypesthesia” OR “numbness” OR “neuropathy” OR “neuropathies” OR “neuropathic” OR “painful post-traumatic trigeminal neuropathy” OR “Neuropathic Pain” OR “Atypical Neuralgia” OR “Atypical Neuralgias” OR “Mandibular Nerve Injuries” OR “Trigeminal Nerve Diseases” OR “Cranial Nerve Injuries” OR “Trigeminal Nerve Injuries” OR “trigeminal neuralgia” OR “Facial Pain” OR “Facial Neuralgia” OR “Facial Nerve Injuries” OR “Glossopharyngeal Nerve Diseases” OR “idiopathic pain”)) AND TS=(“mandibular canal” OR “inferior alveolar nerve” OR “mental nerve” OR “mandibular nerve” OR “maxillary nerve” OR “palatine nerve” OR “infraorbital nerve” OR “facial nerve” OR “trigeminal nerve” OR “mandibular branch” OR “mandibular branches” OR “maxillary branch” OR “maxillary branches” OR “maxillary branch”)) |
| Grey Literature | |
| Google Scholar | (“Dental Implants” OR “Dental Implantation” OR “Dental Implant”) AND (“altered sensation” OR “sensory alterations” OR “sensory disturbance” OR “sensory disturbances” OR “neurosensory disturbance” OR “neuropathy” OR “neuropathies” OR “neuropathic”) |
| Open Grey | (“Dental Implants” OR “Dental Implantation” OR “Dental Implant”) AND (“disturbance” OR “disturbances” OR “pain” OR “injury” OR “injuries” OR “injured” OR “damage” OR “damages” OR “hurt” OR “altered sensation” OR “sensory alterations” OR “sensory disturbance” OR “sensory disturbances” OR “neurosensory disturbance” OR “neurosensory disturbances” OR “allodynia” OR “hyperalgesia” OR “paresthesia” OR “dysesthesia” OR “hypoesthesia” OR “hypesthesia” OR “numbness”) |
| Proquest Dissertations and Thesis | noft(“Dental Implants” OR “Dental Implantation” OR “Dental Implant”) AND noft(“disturbance” OR “disturbances” OR “pain” OR “injury” OR “injuries” OR “injured” OR “damage” OR “damages” OR “hurt” OR “altered sensation” OR “sensory alterations” OR “sensory disturbance” OR “sensory disturbances” OR “neurosensory disturbance” OR “neurosensory disturbances” OR “allodynia” OR “hyperalgesia” OR “paresthesia” OR “dysesthesia” OR “hypoesthesia” OR “hypesthesia” OR “numbness” OR “neuropathy” OR “neuropathies” OR “neuropathic” OR “painful post-traumatic trigeminal neuropathy” OR “Neuropathic Pain” OR “Atypical Neuralgia” OR “Atypical Neuralgias” OR “Mandibular Nerve Injuries” OR “Trigeminal Nerve Diseases” OR “Cranial Nerve Injuries” OR “Trigeminal Nerve Injuries” OR “trigeminal neuralgia” OR “Facial Pain” OR “Facial Neuralgia” OR “Facial Nerve Injuries” OR “Glossopharyngeal Nerve Diseases” OR “idiopathic pain”) |

Supplementary Table 2. Excluded articles and reasons for exclusion (n = 63).

| Author, year | Reason for exclusion |
| --- | --- |
| Agbaje *et al*. [1], 2016 | 4 |
| Amadou-Diaw, Braud and Boucher, 2022 [2] | 3 |
| Al-Khabbaz, Griffin & Al-Shammari, 2007 [3] | 2 |
| Bagheri & Meyer, 2013 [4] | 1 |
| Benevides, De Moraes & De Moraes, 2019 [5] | 1 |
| Bozkaya *et al.* [6], 2014 | 1 |
| Brook, 2012 [7] | 1 |
| Campos *et al.* [8], 2013 | 1 |
| Carter *et al.* [9], 2016 | 4 |
| Chaushu *et al*. [10], 2002 | 3 |
| Choi *et al*. [11], 2014 | 3 |
| Deppe *et al*. [12], 2015 | 4 |
| Devine, Taylor & Renton, 2016 [13] | 2 |
| Diakonoff and Moreau, 2022 [14] | 3 |
| Doustkam *et al*. [15], 2017 | 3 |
| Elias, 1994 [16] | 1 |
| Esposito *et al*. [17], 2014 | 3 |
| Felice *et al*. [18], 2014 | 3 |
| Franco *et al*. [19], 2016 | 1 |
| Froum *et al*. [20], 2023 | 5 |
| Garcia-Blanco *et al*. [21], 2021 | 4 |
| Givol *et al*. [22], 2013 | 3 |
| Göçmen and Bayram, 2023 [23] | 3 |
| Hakimiha *et al*. [24], 2020 | 4 |
| Hillerup, 2007 [25] | 4 |
| Hillerup, 2008 [26] | 4 |
| Ishigaki *et al.* [27], 2009 | 1 |
| Ishigaki, Ono & Yatani, 2014 [28] | 5 |
| Jinno *et al*. [29], 1994 | 1 |
| Kalladka *et al*. [30], 2008 | 4 |
| Kang *et al*. [31], 2021 | 4 |
| Karabit & Kattan, 2018 [32] | 3 |
| Karanam and Krishna, 2022 [33] | 4 |
| Khawaja & Renton, 2009 [34] | 1 |
| Kim & Kim, 2019 [35] | 3 |
| Kim *et al*. [36], 2015 | 3 |
| Klazen *et al*. [37], 2018 | 4 |
| Kumar, Ganapathy & Visalakshi, 2019 [38] | 3 |
| Libersa, Savignat & Tonnel, 2007 [39] | 4 |
| Lim *et al*. [40], 2017 | 1 |
| Martínez-González *et al*. [41], 2011 | 3 |
| Miloro & Criddle, 2018 [42] | 4 |
| Na *et al*. [43], 2019 | 3 |
| Oliveira, 2018 [44] | 4 |
| Pääsky, Suomalainen & Ventä, 2021 [45] | 5 |
| Peñarrocha *et al*. [46], 2012 | 4 |
| Perea-Pérez *et al*. [47], 2014 | 3 |
| Politis *et al*. [48], 2017 | 2 |
| Progrel *et al*. [49], 2011 | 6 |
| Renton & Devine, 2013 [50] | 1 |
| Renton & Yilmaz, 2011 [51] | 4 |
| Renton *et al*. [52], 2012 | 3 |
| Sanner *et al*. [53], 2022 | 3 |
| Scarano *et al*. [54], 2017 | 3 |
| Sharma *et al*. [55], 2022 | 3 |
| Shin *et al*. [56], 2021 | 5 |
| Tay & Zuniga, 2007 [57] | 4 |
| Tufekcioglu *et al*. [58], 2015 | 6 |
| Vázquez-Delgado *et al*. [59], 2018 | 3 |
| von Eckardstein, Keil & Rohde, 2015 [60] | 3 |
| Walton, 2000 [61] | 7 |
| Wismeijer *et al*. [62], 1997 | 7 |
| Yates & Zuniga, 2012 [63] | 1 |

1—Book chapters, conference abstracts, expert opinion, letters, literature reviews, study protocol, magazine section, and case series with less than five patients; 2—Non-neuropathic pain postoperative assessment; 3—Evaluation other outcomes than prevalence and treatment; 4—Evaluation of neuropathic pain and/or nerve injury after procedures other than dental implant; 5—Full-text not available; 6—Incomplete data; 7—Presence of nerve injury and/or neuropathies before the implant surgery.

References

[1] Agbaje JO, Van de Casteele E, Hiel M, Verbaanderd C, Lambrichts I, Politis C. Neuropathy of trigeminal nerve branches after oral and maxillofacial treatment. Journal of Oral and Maxillofacial Surgery. 2016; 15: 321–327.

[2] Amadou-Diaw N, Braud A, Boucher Y. Persistent, neuropathic-like trigeminal pain after dental implant loading. Journal of Clinical and Experimental Dentistry. 2022; 14: e185–e191.

[3] Al-Khabbaz AK, Griffin TJ, Al-Shammari KF. Assessment of pain associated with the surgical placement of dental implants. Journal of Periodontology. 2007; 78: 239–246.

[4] Bagheri SC, Meyer RA. Dental implant-related injuries of the trigeminal nerve. In: Miloro M (ed). Trigeminal Nerve Injuries Chicago: Springer. 2013: 87–107.

[5] Benevides LE, De Moraes EJ, De Moraes NB. Incidence of peripheral nerve injury in oral maxillofacial surgery: a restrospective clinical study. Journal of Oral and Maxillofacial Surgery. 2019; 48: 60.

[6] Bozkaya S, Cakir M, Peker E, Ogutlu F. Effect of low-level laser therapy on neurosensory recovery after dental procedures. Journal of Oral and Maxillofacial Surgery. 2014; 72: e119–e120.

[7] Brook I. Summary of: Post-implant neuropathy of the trigeminal nerve. A case series. British Dental Journal. 2012; 212: 544–545.

[8] Campos CG, Piccoli AP, Marson FC, Anjos Neto-Filho Md, Lolli LF, Silva CO. Subjective assessment of inferior alveolar nerve function after lateralization surgery. Dental Press Implantology. 2013; 7: 71–78.

[9] Carter E, Yilmaz Z, Devine M, Renton T. An update on the causes, assessment and management of third division sensory trigeminal neuropathies. British Dental Journal. 2016; 220: 627–635.

[10] Chaushu G, Taicher S, Halamish-Shani T, Givol N. Medicolegal aspects of altered sensation following implant placement in the mandible. The International Journal of Oral & Maxillofacial Implants. 2002; 17: 413–415.

[11] Choi YC, Cho ES, Merrill RL, Kim ST, Ahn HJ. Analysis of neurosensory dysfunction after dental implant surgery. Journal of Oral Medicine and Pain. 2014; 39: 133–139.

[12] Deppe H, Mucke T, Wagenpfeil S, Kesting M, Linsenmeyer E, Tolle T. Trigeminal nerve injuries after mandibular oral surgery in a university outpatient setting-a retrospective analysis of 1,559 cases. Clinical Oral Investigations. 2015; 19: 149–157.

[13] Devine M, Taylor S, Renton T. Chronic post-surgical pain following the placement of dental implants in the maxilla: A case series. European Journal of Oral Implantology. 2016; 9: 179–186.

[14] Diakonoff H, Moreau N. Inferior alveolar nerve injury following dental implant placement: a medicolegal analysis of French liability lawsuits. Journal of Stomatology, Oral and Maxillofacial Surgery. 2022; 123: 158–162.

[15] Doustkam AA, Quang SV, Lescaille G, Descroix V. Chronic neuropathic pain following dental implant surgery: report of 8 cases. Medecine Buccale Chirurgie Buccale. 2017; 23: 13–19.

[16] Elias AC. Prevalence of altered sensations associated with implant surgery. The International Journal of Oral & Maxillofacial Implants. 1994; 9: 146.

[17] Esposito M, Pistilli R, Barausse C, Felice P. Three-year results from a randomised controlled trial comparing prostheses supported by 5-mm long implants or by longer implants in augmented bone in posterior atrophic edentulous jaws. European Journal of Oral Implantology. 2014; 7: 383–395.

[18] Felice P, Cannizzaro G, Barausse C, Pistilli R, Esposito M. Short implants versus longer implants in vertically augmented posterior mandibles: a randomised controlled trial with 5-year after loading follow-up. European Journal of Oral Implantology. 2014; 7: 359–369.

[19] Franco A, Fernandes MM, Kichler A, Rodriguez JCZ, Oliveira RN. Computed tomography as legal evidence of malpractice in dentomaxillofacial Implantology: report of a case series. Revista Odontológica do Brasil Central. 2016; 25: 12–15.

[20] Froum SJ, Reis N, Kukiratirat T, De La Torre EG, Barsoum A, Yu PYC, *et al*. A retrospective study of implant placement lateral to the inferior alveolar nerve (ILIAN) in severely atrophic posterior mandibular ridges. International Journal of Periodontics and Restorative Dentistry. 2023; 43: E173–E180.

[21] Garcia-Blanco M, Gualtieri AF, Lovaglio-Rivas AC, Ruffini JM, Puia SA. Trigeminal nerve injuries. Four years’ experience at a single Argentine referral center and a literature review. Acta Odontológica Latinoamericana. 2021; 34: 263–270.

[22] Givol N, Peleg O, Yarom N, Blinder D, Lazarovici TS. Inferior alveolar neurosensory deficiency associated with placement of dental implants. Journal of Periodontology. 2013; 84: 495–501.

[23] Göçmen G, Bayram F. Evaluating the influence of the mandibular canal trajectory on the duration of postoperative paraesthesia in patients undergoing inferior alveolar nerve lateralisation: a prospective cohort study. British Journal of Oral and Maxillofacial Surgery. 2023; 61: 540–546.

[24] Hakimiha N, Rokn AR, Younespour S, Moslemi N. Photobiomodulation therapy for the management of patients with inferior alveolar neurosensory disturbance associated with oral surgical procedures: an interventional case series study. Journal of Lasers in Medical Sciences. 2020; 11: s113–s118.

[25] Hillerup S. Iatrogenic injury to oral branches of the trigeminal nerve: records of 449 cases. Clinical Oral Investigations. 2007; 11: 133–142.

[26] Hillerup S. Iatrogenic injury to the inferior alveolar nerve: etiology, signs and symptoms, and observations on recovery. International Journal of Oral and Maxillofacial Surgery. 2008; 37: 704–709.

[27] Ishigaki S, Ono K, Miyauchi T, Yatani H. The effect of the implant surgery on the pain threshold of the trigeminal nerve region. European Journal of Pain. 2009; 13: S127.

[28] Ishigaki S, Ono K, Yatani H. Time course study of postoperative pain after dental implant surgery. 7th World Congress. Switzerland: World Institute of Pain. 2014.

[29] Jinno S, Ito H, Fukayama H, Jirose Y, Kubota Y. A retrospective study of trigeminal paresthesia after dental treatment. Journal of Japanese Dental Society of Anesthesiology. 1994; 22: 466–474.

[30] Kalladka M, Proter N, Benoliel R, Czerninski R, Eliav E. Mental nerve neuropathy: patient characteristics and neurosensory changes. Oral Surgery, Oral Medicine, Oral Pathology, and Oral Radiology 2008; 106: 364–370.

[31] Kang SK, Almansoori AA, Chae YS, Kim B, Kim SM, Lee JH. Factors affecting functional sensory recovery after inferior alveolar nerve repair using the nerve sliding technique. Journal of Oral and Maxillofacial Surgery. 2021; 79: 1794–1800.

[32] Karabit ZZ, Al Kattan FZ. Is there any relationship between the inferior alveolar nerve injuries caused by implant surgery and the qualification of the dentist? Cross-sectional study. New Armenian Medical Journal. 2018; 12: 29–35.

[33] Karanam A, Krishna T. Assessment of neuropathy of trigeminal nerve branches after oral and maxillofacial treatment. NeuroQuantology. 2022; 20: 7794–7798.

[34] Khawaja N, Renton T. Case studies on implant removal influencing the resolution of inferior alveolar nerve injury. British Dental Journal. 2009; 206: 365–370.

[35] Kim HK, Kim ME. Quantitative and qualitative sensory testing results are associated with numbness rather than neuropathic pain in patients with post-implant trigeminal neuropathy: a cross-sectional pilot study. Somatosensory & Motor Research. 2019; 36: 202–211.

[36] Kim YK, Yun PY, Kim JH, Lee JY, Lee W. The quantitative sensory testing is an efficient objective method for assessment of nerve injury. Maxillofacial Plastic and Reconstructive Surgery. 2015; 37: 1–7.

[37] Klazen Y, Van der Cruyssen F, Vranckx M, Van Vlierberghe M, Politis C, *et al*. Iatrogenic trigeminal post-traumatic neuropathy: a retrospective two-year cohort study. International Journal of Oral and Maxillofacial Surgery. 2018; 47: 789–793.

[38] Kumar JS, Ganapathy D, Visalakshi RM. Awareness of incidence and management of nerve injuries during implant treatment. Drug Invention Today. 2019; 12: 1032–1035.

[39] Libersa P, Savignat M, Tonnel A. Neurosensory disturbances of the inferior alveolar nerve: a retrospective study of complaints in a 10-year period. Journal of Oral and Maxillofacial Surgery. 2007; 65: 1486–1489.

[40] Lim HK, Byun SH, Kim SM, Kim MJ, Lee JH. Facial pain after dental implant installation on maxilla. International Journal of Oral and Maxillofacial Surgery. 2017; 46: 271.

[41] Martínez-González JM, Martínez-Rodríguez N, Calvo-Guirado JL, Brinkmann JC, Dorado CB. Glossopharyngeal neuralgia: a presentation of 14 cases. Journal of Oral and Maxillofacial Surgery. 2011; 69: e38–41.

[42] Miloro M, Criddle TR. Does low-level laser therapy affect recovery of lingual and inferior alveolar nerve injuries? Journal of Oral and Maxillofacial Surgery. 2018; 76: 2669–2675.

[43] Na JY, Han S-S, Jeon K, Choi YJ, Choi SH, Lee C. Prognosis in case of nerve disturbance after mandibular implant surgery in relation to computed tomography findings and symptoms. Journal of Periodontal & Implant Science. 2019; 49: 127–135.

[44] Oliveira KDCM. Effectiveness of lasertherapy, laseracupuntura and systemic medication for treatment of parestesia in patients who underwent implants and mandibular third molar surgeries. University of Sao Paulo. 2018: 112–112.

[45] Pääsky E, Suomalainen A, Ventä I. Are women more susceptible than men to iatrogenic inferior alveolar nerve injury in dental implant surgery? International Journal of Oral and Maxillofacial Surgery. 2021; 51: 251–256.

[46] Peñarrocha M, Peñarrocha D, Bagán JV, Peñarrocha M. Post-traumatic trigeminal neuropathy. A study of 63 cases. Medicina Oral, Patología Oral y Cirugía Bucal. 2012; 17: e297–300.

[47] Perea-Pérez B, Labajo-González E, Santiago-Sáez A, Albarrán-Juan E, Villa-Vigil A. Analysis of 415 adverse events in dental practice in Spain from 2000 to 2010. Medicina Oral, Patología Oral y Cirugía Bucal. 2014; 19: e500–e505.

[48] Politis C, Agbaje J, Van Hevele J, Nicolielo L, De Laat A, Lambrichts I, *et al*. Report of neuropathic pain after dental implant placement: a case series. The International Journal of Oral & Maxillofacial Implants. 2017; 32: 439–444.

[49] Pogrel MA, Jergensen R, Burgon E, Hulme D. Long-term outcome of trigeminal nerve injuries related to dental treatment. Journal of Oral and Maxillofacial Surgery. 2011; 69: 2284–2288.

[50] Renton T, Devine M. Diagnosis and management of inferior alveolar nerve damage associated with dental implant surgery. Forum Implantologicum. 2013; 9: 16–27.

[51] Renton T, Yilmaz Z. Profiling of patients presenting with posttraumatic neuropathy of the trigeminal nerve. Journal of Oral & Facial Pain and Headache. 2011; 25: 333–344.

[52] Renton T, Dawood A, Shah A, Searson L, Yilmaz Z. Post-implant neuropathy of the trigeminal nerve. A case series. British Dental Journal. 2012; 212: 1–6.

[53] Sanner F, Sonntag D, Hambrock N, Zehnder M. Patients with persistent idiopathic dentoalveolar pain in dental practice. International Endodontic Journal. 2022; 55: 231–239.

[54] Scarano A, Sinjari B, Murmura G, Lorusso F. Neurosensory disturbance of the inferior alveolar nerve after 3025 implant placements. Implant Dentistry. 2017; 26: 735–743.

[55] Sharma R, Modi R, Yadav A, Singh D. Assessment of incidence of inferior alveolar nerve (IAN) lesion and duration of sensitivity disturbances after the insertion of dental implants—an original research. NeuroQuantology. 2022; 20: 5674–5678.

[56] Shin YM, Choi SY, Lee DH, Jung JK, Kwon TG. Management of chronic idiopathic pain in patients with dental implant without a clear pathological lesion: a retrospective study. Journal of Oral Implantology. 2021; 48: 301–306.

[57] Tay ABG, Zuniga JR. Clinical characteristics of trigeminal nerve injury referrals to a university centre. International Journal of Oral and Maxillofacial Surgery. 2007; 36: 922–927.

[58] Tufekcioglu S, Delilbasi C, Gurler G, Dilaver E, Ozer N. Is 2 mm a safe distance from the inferior alveolar canal to avoid neurosensory complications in implant surgery? Nigerian Journal of Clinical Practice. 2015; 20: 274–277.

[59] Vázquez-Delgado E, Viaplana-Gutiérrez M, Figueiredo R, Renton T, Gay-Escoda C, Valmaseda-Castellón E. Prevalence of neuropathic pain and sensory alterations after dental implant placement in a university-based oral surgery department: a retrospective cohort study. Gerodontology. 2018; 35: 117–122.

[60] von Eckardstein KL, Keil M, Rohde V. Unnecessary dental procedures as a consequence of trigeminal neuralgia. Neurosurgical Review. 2015; 38: 355–360.

[61] Walton JN. Altered sensation associated with implants in the anterior mandible: a prospective study. Journal of Prosthetic Dentistry. 2000; 83: 443–449.

[62] Wismeijer D, vanWaas MAJ, Vermeeren J, Kalk W. Patients’ perception of sensory disturbances of the mental nerve before and after implant surgery: a prospective study of 110 patients. British Journal of Oral and Maxillofacial Surgery. 1997; 35: 254–259.

[63] Yates D, Zuniga J. Factors associated with the recurrence or resolution of neuropathic painfollowing trigeminal nerve repair for neuropathic pain. Journal of Oral and Maxillofacial Surgery. 2012; 70: e15–e16.

Supplementary Table 3. Neuropathies’ prevalence data according to analysis times after dental implant surgery (if applicable).

Supplementary Table 3A. Prevalence of neuropathies after dental implant placement without nerve lateralization (outcome 1).

| Study | Total sample | Until one week | More than three months |
| --- | --- | --- | --- |
| Abarca *et al.* [29] 2006 | 58 | NR | 32.75% |
| Bartling, Freeman & Kraut 1999 | 94 | 8.51% | 4.25% |
| Dannan, Alkattan & Jackowaski 2013 | 19 | 21.05% | 15.79% |
| Ellies & Hawker 1993 | 87 | 18.39% | 4.60% |
| Felice *et al.* [34] 2009 | 15 | 13% | 0% |
| Felice *et al.* [25] 2009 | 60 | 30% | 0% |
| Filipov *et al.* [21] 2023 | 9 | 22.22% | 11.11% |
| Hartmann, Welte-Jzyk & Seiler 2017 | 60 | 0% | 0% |
| Porporatti *et al.* [27] 2017 | 20 | 0% | 0% |
| Tejada *et al.* [15] 2021 | 225 | NR | 8.88% |

NR: not reported.

Supplementary Table 3B. Prevalence of neuropathies after dental implant placement with nerve lateralization (outcome 2).

| Study | Total sample | Until one week | More than three months |
| --- | --- | --- | --- |
| Al-Almaie *et al.* [20], 2020 | 10 | 60% | 20% |
| Atef & Mounir, 2018 | 7 | 85.71% | 0% |
| Castellano-Navarro *et al.* [18], 2019 | 139 | 94.96% | 70.51% |
| de Campos *et al.* [17], 2019* | 34 | 100% | 50% |
| Deryabin & Grybauskas, 2021 | 23 | 100% | 65.22% |
| Díaz & Gías, 2013 | 19 | 100% | NR |
| Di Pillo & Ropoport, 2009 | 12 | 91.66% | 75% |
| Ferrigno, Laureti & Fanali, 2005 | 19 | 52.63% | 21.05% |
| Friberg, Ivanoff & Lekholm, 1992 | 10 | 100% | 50% |
| Hashemi, 2010 | 110 | 100% | 2.72% |
| Hori *et al.* [43], 2001 | 8 | 100% | 87.5% |
| Kan *et al.* [44], 1997 | 21 | NR | 52.38% |
| Khojasteh *et al.* [45], 2016* | 14 | NR | 92.86% |
| Lorean *et al.* [46], 2013* | 57 | 7.02% | NR |
| Martínez-Rodríguez *et al.* [47], 2016 | 27 | 100% | 25.93% |
| Mavriqi, Mortellaro & Scarano, 2016 | 12 | NR | 8.33% |
| Morrison, Chiarot & Kirby, 2002 | 20 | NR | 25% |
| Nishimaki *et al.* [50], 2016 | 7 | 100% | 71.43% |
| Peleg *et al.* [51], 2002* | 10 | 60% | 0% |
| Rathod *et al.* [52], 2019* | 10 | 100% | 80% |

NR: not reported; *Articles in which the samples are patients, and not surgery sites.

Supplementary Table 4. Methodological quality of included studies according Joanna Briggs Institute critical appraisal tools.

Supplementary Table 4A. Case series (n = 20).

|  | Q1 | Q2 | Q3 | Q4 | Q5 | Q6 | Q7 | Q8 | Q9 | Q10 | Quality |
| --- | --- | --- | --- | --- | --- | --- | --- | --- | --- | --- | --- |
| Abarca *et al.* [29], 2006 | Y | Y | Y | Y | Y | Y | Y | NA | NA | U | High |
| Al-Amaie *et al.* [20], 2020 | N | Y | Y | Y | Y | Y | Y | NA | NA | U | Moderate |
| Bartling *et al.* [30], 1999 | Y | Y | Y | Y | Y | Y | Y | NA | NA | U | High |
| Castellano-Navarro *et al.* [18], 2019 | Y | Y | N | N | N | Y | Y | NA | NA | N | Low |
| Dannan et al., 2013 | Y | Y | Y | Y | Y | Y | Y | NA | NA | Y | High |
| Deryabin & Grybauskas, 2021 | Y | Y | N | Y | Y | Y | Y | NA | NA | N | Moderate |
| Ellies & Hawker *et al.* [31], 1993 | Y | Y | Y | Y | Y | Y | Y | NA | NA | Y | High |
| Filipov *et al.* [21], 2023 | Y | Y | Y | Y | Y | N | Y | NA | NA | Y | High |
| Garcia-Blanco *et al.* [4], 2017 | Y | U | U | Y | Y | Y | Y | NA | NA | Y | Moderate |
| Hashemi 2010, | Y | Y | N | Y | Y | Y | Y | NA | NA | N | Moderate |
| Hori *et al.* [43], 2001 | N | Y | N | Y | Y | Y | Y | NA | NA | N | Low |
| Kan *et al.* [44], 1997 | N | Y | Y | Y | Y | Y | Y | NA | NA | N | Moderate |
| Khojasteh *et al.* [45], 2016 | Y | Y | Y | Y | Y | Y | Y | NA | NA | Y | High |
| Lorean *et al.* [46], 2013 | N | Y | Y | Y | Y | Y | Y | NA | NA | N | Moderate |
| Martínez-Rodríguez *et al.* [47], 2016 | Y | Y | Y | Y | Y | Y | Y | NA | NA | Y | High |
| Mavriqi, Mortellaro & Scarano, 2016 | N | Y | Y | Y | Y | Y | Y | NA | NA | N | Moderate |
| Morrison, Chiarot & Kirby, 2002 | Y | Y | Y | Y | Y | Y | Y | NA | NA | Y | High |
| Peleg *et al.* [51], 2002 | N | Y | N | N | N | Y | Y | NA | NA | N | Low |
| Tejada *et al.* [15], 2021 | Y | Y | Y | Y | Y | Y | Y | NA | NA | Y | High |
| Vazquez *et al.* [33], 2008 | Y | Y | N | Y | Y | Y | Y | NA | NA | Y | High |

Q1: Were there clear criteria for inclusion in the case series?

Q2: Was the condition measured in a standard, reliable way for all participants included in the case series?

Q3: Were valid methods used for identification of the condition for all participants included in the case series?

Q4: Did the case series have consecutive inclusion of participants?

Q5: Did the case series have complete inclusion of participants?

Q6: Was there clear reporting of the demographics of the participants in the study?

Q7: Was there clear reporting of clinical information of the participants?

Q8: Were the outcomes or follow up results of cases clearly reported?

Q9: Was there clear reporting of the presenting site(s)/clinic(s) demographic information?

Q10: Was statistical analysis appropriate?

Supplementary Table 4B. Before-and-after and not randomized trial (n = 16).

|  | Q1 | Q2 | Q3 | Q4 | Q5 | Q6 | Q7 | Q8 | Q9 | Quality |
| --- | --- | --- | --- | --- | --- | --- | --- | --- | --- | --- |
| Atef & Mounir, 2018 | Y | Y | Y | NA | N | Y | Y | Y | Y | High |
| Díaz & Gías, 2013 | Y | Y | Y | NA | N | Y | Y | N | Y | Moderate |
| Di Pillo & Rapoport, 2009 | Y | Y | Y | NA | N | Y | Y | N | Y | Moderate |
| Felice *et al.* [34], 2009 A | Y | Y | Y | N | N | Y | Y | Y | Y | Moderate |
| Felice *et al.* [35], 2009 B | Y | Y | Y | N | N | Y | Y | Y | Y | Moderate |
| Ferrigno, Laureti & Fanali, 2005 | Y | Y | Y | NA | N | Y | Y | N | Y | Moderate |
| Friberg, Ivanoff & Lekholm, 1992 | Y | Y | Y | NA | N | Y | Y | N | Y | Moderate |
| Gagik *et al.* [53], 2020 | Y | Y | Y | Y | N | Y | Y | N | Y | Moderate |
| Hartmann, Welte-Jzyk & Seiler, 2017 | Y | Y | Y | Y | Y | Y | Y | Y | Y | High |
| Juodzbalys *et al.* [59], 2011 | Y | Y | Y | NA | Y | Y | Y | Y | Y | High |
| Kim *et al.* [54], 2013 | Y | Y | Y | NA | Y | Y | Y | N | Y | High |
| Nishimaki *et al.* [50], 2016 | Y | Y | Y | NA | N | Y | Y | N | Y | Moderate |
| Park, Lee, and Kim, 2010 | Y | Y | Y | NA | Y | Y | Y | N | Y | High |
| Porporatti, 2006; Porporatti *et al.* [27], 2017 | Y | Y | Y | NA | Y | Y | Y | Y | Y | High |
| Rathod *et al.* [52], 2019 | Y | Y | Y | NA | Y | Y | Y | N | Y | High |

Q1: Is it clear in the study what is the “cause” and what is the “effect” (*i.e.*, there is no confusion about which variable comes first)?

Q2: Were the participants included in any comparisons similar?

Q3: Were the participants included in any comparisons receiving similar treatment/care, other than the exposure or intervention of interest?

Q4: Was there a control group?

Q5: Were there multiple measurements of the outcome both pre and post the intervention/exposure?

Q6: Was follow up complete and if not, were differences between groups in terms of their follow up adequately described and analyzed?

Q7: Were the outcomes of participants included in any comparisons measured in the same way?

Q8: Were outcomes measured in a reliable way?

Q9: Was appropriate statistical analysis used?

Supplementary Table 4C. Randomized controlled trials (n = 2).

|  | Q1 | Q2 | Q3 | Q4 | Q5 | Q6 | Q7 | Q8 | Q9 | Q10 | Q11 | Q12 | Q13 | Quality |
| --- | --- | --- | --- | --- | --- | --- | --- | --- | --- | --- | --- | --- | --- | --- |
| de Campos *et al.* [17], 2019 | Y | Y | Y | Y | N | U | Y | Y | N | Y | U | Y | Y | Low |
| Ghasemi *et al.* [57], 2022 | Y | Y | Y | Y | N | N | Y | Y | N | Y | Y | Y | Y | Moderate |

Q1: Was true randomization used for assignment of participants to treatment groups?

Q2: Was allocation to treatment groups concealed?

Q3: Were treatment groups similar at the baseline?

Q4: Were participants blind to treatment assignment?

Q5: Were those delivering treatment blind to treatment assignment?

Q6: Were outcomes assessors blind to treatment assignment?

Q7: Were treatment groups treated identically other than the intervention of interest?

Q8: Was follow up complete and if not, were differences between groups in terms of their follow up adequately described and analyzed?

Q9: Were participants analyzed in the groups to which they were randomized?

Q10: Were outcomes measured in the same way for treatment groups?

Q11: Were outcomes measured in a reliable way?

Q12: Was appropriate statistical analysis used?

Q13: Was the trial design appropriate, and any deviations from the standard RCT design (individual randomization, parallel groups) accounted for in the conduct and analysis of the trial?
